# Unveiling the potentials of biocompatible silver nanoparticles on human lung carcinoma A549 cells and *Helicobacter pylori*

**DOI:** 10.1038/s41598-019-42112-1

**Published:** 2019-04-08

**Authors:** Kandasamy Saravanakumar, Ramachandran Chelliah, Davoodbasha MubarakAli, Deog-Hwan Oh, Kandasamy Kathiresan, Myeong-Hyeon Wang

**Affiliations:** 1 Department of Medical Biotechnology, College of Biomedical Sciences, Kangwon National University, Chuncheon, Gangwon 24341 Republic of Korea; 2 Department of Food Science and Biotechnology College of Biotechnology and Bioscience, Kangwon National University, Chuncheon, Republic of Korea; 3 School of Life Sciences, B.S. Abdur Rahman Crescent Institute of Science and Technology, Chennai, 600048 India; 4 Centre of Advanced Study in Marine Biology, Faculty of Marine Sciences, Annamalai University, Parangipettai, 608 502 Tamil Nadu India

## Abstract

Silver nanoparticles (AgNPs) are gaining importance in health and environment. This study synthesized AgNPs using the bark extract of a plant, *Toxicodendron vernicifluum* (Tv) as confirmed by a absorption peak at 420 nm corresponding to the Plasmon resonance of AgNPs. The AgNPs were spherical, oval-shaped with size range of 2–40 nm as evident by field emission transmission electron microscopy (FE-TEM) and particle size analysis (PSA). The particles formed were crystalline by the presence of (111), (220) and (200) planes, as revealed by X ray diffraction (XRD) and energy dispersive spectroscopy (EDS). The presence of amine, amide, phenolic, and alcoholic aromatics derived from Tv extract was found to be capping and or reducing agents as evident by Fourier-transform infrared spectroscopy (FTIR) spectra. The Tv-AgNPs were observed to be biocompatible to chick embryonic and NIH3T3 cells at various concentrations. Interestingly, Tv-AgNPs at the concentration of 320 µg. mL^−1^ induced 82.5% of cell death in human lung cancer, A549 cells and further 95% of cell death with annexin V FITC/PI based apoptosis. The Tv-AgNPs selectively targeted and damaged the cancer cells through ROS generation. The Tv-AgNPs displayed minimal inhibitory concentration (MIC) of 8.12 µg.mL^−1^ and 18.14 µg.mL^−1^ against STEC and *H. pylori* respectively. This multi-potent property of Tv-AgNPs was due to shape and size specific property that facilitated easy penetration into the bacterial and cancer cells for targeted therapy.

## Introduction

Phytochemicals-medicated synthesis of metal nanoparticles has received due attention because of their bioactivities such as antibiotic, cytotoxic, drug cargo and photocatalytic potentials^1,2^. Among the metallic nanomaterials, silver nanoparticles (AgNPs) are of significance for their antibacterial effect on human pathogens^3–5^, wound healing^6^, antioxidant^7^, anticancer activities, and dental applications as acrylic resins, composite resins and adhesives, endodontics, periodontal materials, porcelain restoration, titanium implants, and orthodontics^8^. The surgical sutures, when coated with AgNPs are shown to prevent the post wound healing infections^9^. The potent antimicrobial properties of AgNPs has increased the demand in medical applications. AgNPs-based medical products are also available in market such as contraceptive devices, bone prostheses, biomedical devices, wound dressing, and surgical instruments^10–13^.

The multi-drug resistant pathogens are causing the life-threatening human diseases. In this regard, the Gram-negative *Helicobacter pylori* colonizes the gastric epithelium, and it causes several illnesses and chronic diseases in human^14^. This pathogen is known to produce urease enzyme, which converts the urea to ammonia and bicarbonate resulting in neutralization of acidic pH in stomach to create appropriate pH (4.5–7.0) for pathogenic colonization^15^. The eradication of *H. pylori* can prevent various gastrointestinal diseases including peptic ulcer, gastritis, mucosa-associated lymphoid lymphoma, and adenocarcinoma^16^. Recent advancement in the nanotechnology has developed several drug delivery systems to target *H. pylori*^17–21^. For the instance, the amoxillin loaded in the PLGA (poly(lactic-co-glycolic acid) functionalized with receptor UreI has enhanced targeted drug delivery towards eradication of *H. pylori*^22^. Another Gram-negative bacterium is Shiga toxin (Stx1 and Stx2) producing *Escherichia coli* (STEC), colonising in the human gut and causing Hemolytic uremic syndrome (HUS), hemorrhagic colitis, pneumonia, urinary infections, meningitis, and bacteremia, diarrhea^23^.

Environmental pollution and cigarette smoking habits have significantly increased the incidence of lung and cardiovascular diseases^24,25^. Chemotherapeutics, radiation, and surgical approaches for curing the diseases are expensive, often toxic to normal cells and also causing side effects^1,26^. In this context, AgNPs are advantageous in eliciting cancer cell death through the cell cycle arrest, mitochondrial pathways (Reactive oxygen species (ROS) generation), nucleus damage, apoptosis through up-regulation or down-regulation of apoptosis pathways related proteins and genes, necrosis, DNA damage, autophagy and oxidative stress^27,28^, Therefore, fabrication of biocompatible nanoparticles with no side effects can be helpful in successful treatment of the cancer cells.

Green synthesize of AgNPs using the plant extracts are potentially less in toxicity to normal cells, ecologically sustainable, economically viable and less time consuming approach^29^. Several reports are available on the synthesis of AgNPs from plants such as *Trapa natans*^27^*, Phoenix dactylifera*^30^, *Cleome viscosa L*.^31^, *Lycium chinense*^32^, *Taxus baccata*^33^, *Clerodendrum phlomidis*^34^ and their cytotoxicity on cancer cells. The AgNPs synthesized from *Rhus coriaria* under the genus *Rhus* (*Toxicodendron*) and the family *Anacardiaceae* are reported for cytotoxicity on human breast cancer cell line (MCF-7). Another species of the same genus and family is *T. vernicifluum*, reported to have antitumorigenic, antioxidant, neuroprotective, and cytotoxicity effects^35–37^ but not used for the synthesis of AgNPs. Hence, the present work synthesised the silver nanoparticles (Tv-AgNPs) using the aqueous extract of bark derived from *T. vernicifluum* and characterized using the FE-TEM-EDS, PSA, FTIR, and XRD. Antibacterial, cytotoxic and anti-proliferation activities of Tv-AgNPs were also investigated.

## Results and Discussion

### Phytogenic silver nanoparticles

**Figure 1:**
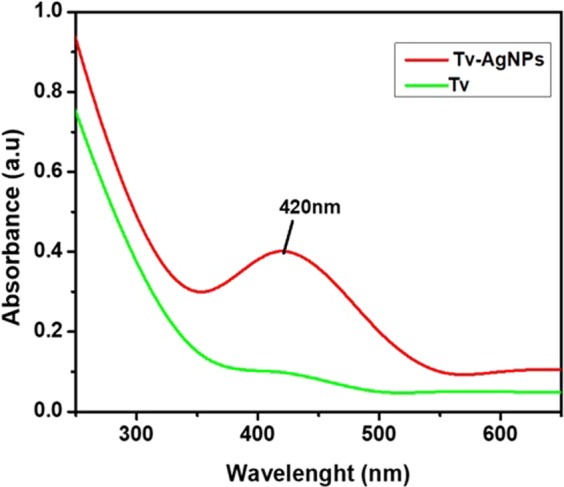
Ultraviolet-vis spectrum of Tv –AgNPs synthesized by the reaction of 3 mM AgNO_3_ and 5 ml of aqueous bark extract from *T. vernicifluum.*

**Figure 2:**
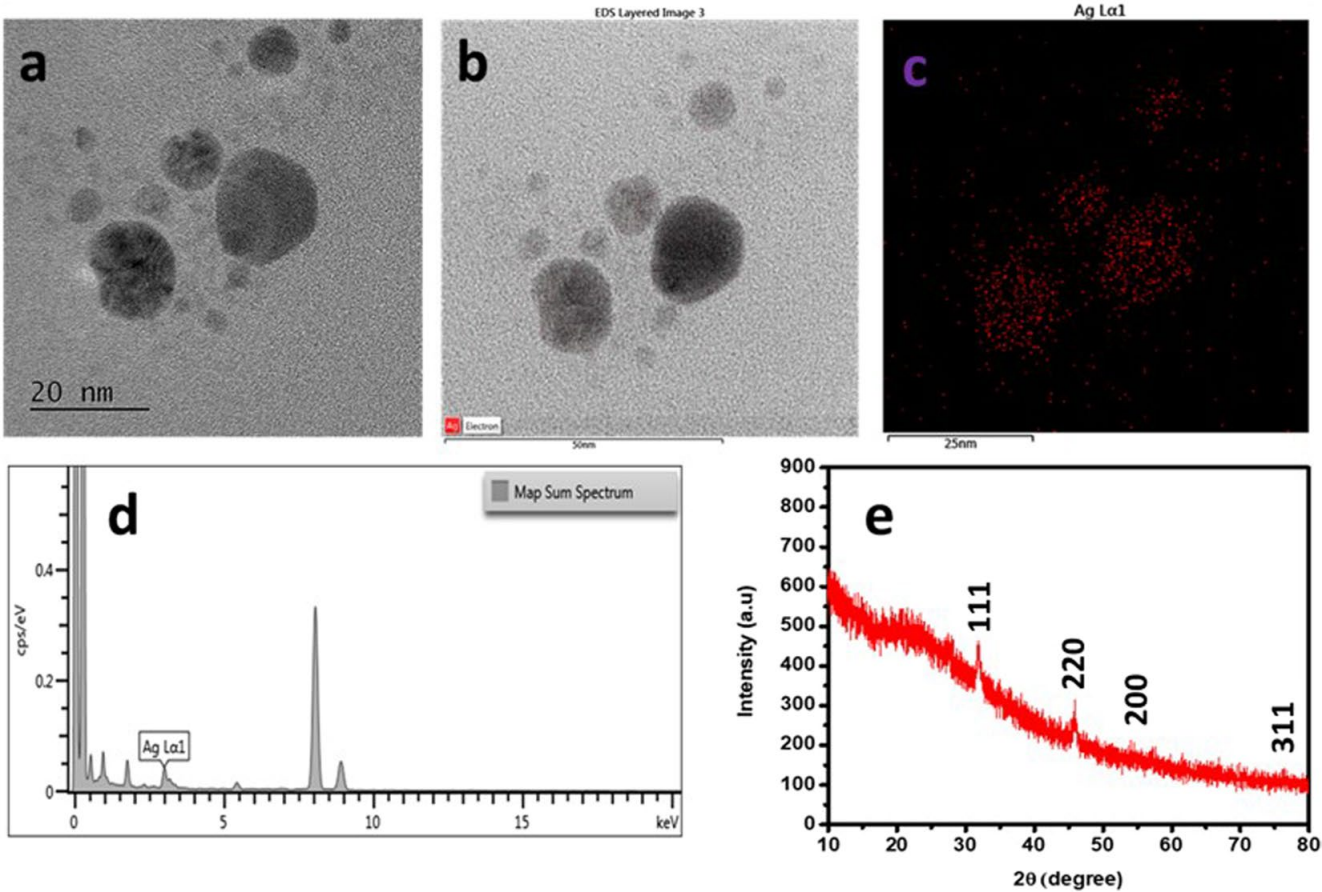
Field-Emission Transmission Electron Microscope (FE-TEM) observation of Tv –AgNPs synthesized by aqueous bark extract from *T. vernicifluum*. Anisotropic structured of Tv-AgNPs visualized by FE-TEM (**a**), scanning of the Ag^+^ in FETEM micrographs by Energy dispersive X-ray spectroscopic (EDS) (**b,c**) Determination of the Ag^+^ by FETEM-EDS spectra (**d**) XRD pattern of Tv-AgNPs (**e**).

Biochemical substances including phenolics, and flavonoids from the plants act as reducing or capping agent for reduction of the silver ions, facilitating the phytogenic synthesis of AgNPs. This was confirmed through absorption plasmon resonance ranged from 400–450 nm by UV-vis spectrophotometer^38^, as well as through observation of color changes from pale yellow to brown colour in the reaction mixture after 12 hours of incubation as also indicated by UV-vis absorption peak at 420 nm corresponding to the AgNPs plasmon resonance (Fig. 1). Further, the FETEM analysis was made to study the morphology, size, and shape of Tv-AgNPs and the results revealed the Tv-AgNPs as anisotropic in structure, spherical and oval-shaped with size range of 2–40 nm (Fig. 2a). In addition, the FETEM-EDS based mapping and chromatographs indicated the presence of Ag in micrographs (Fig. 2b–d), in accordance with the previous reports^39–41^.

**Figure 3:**
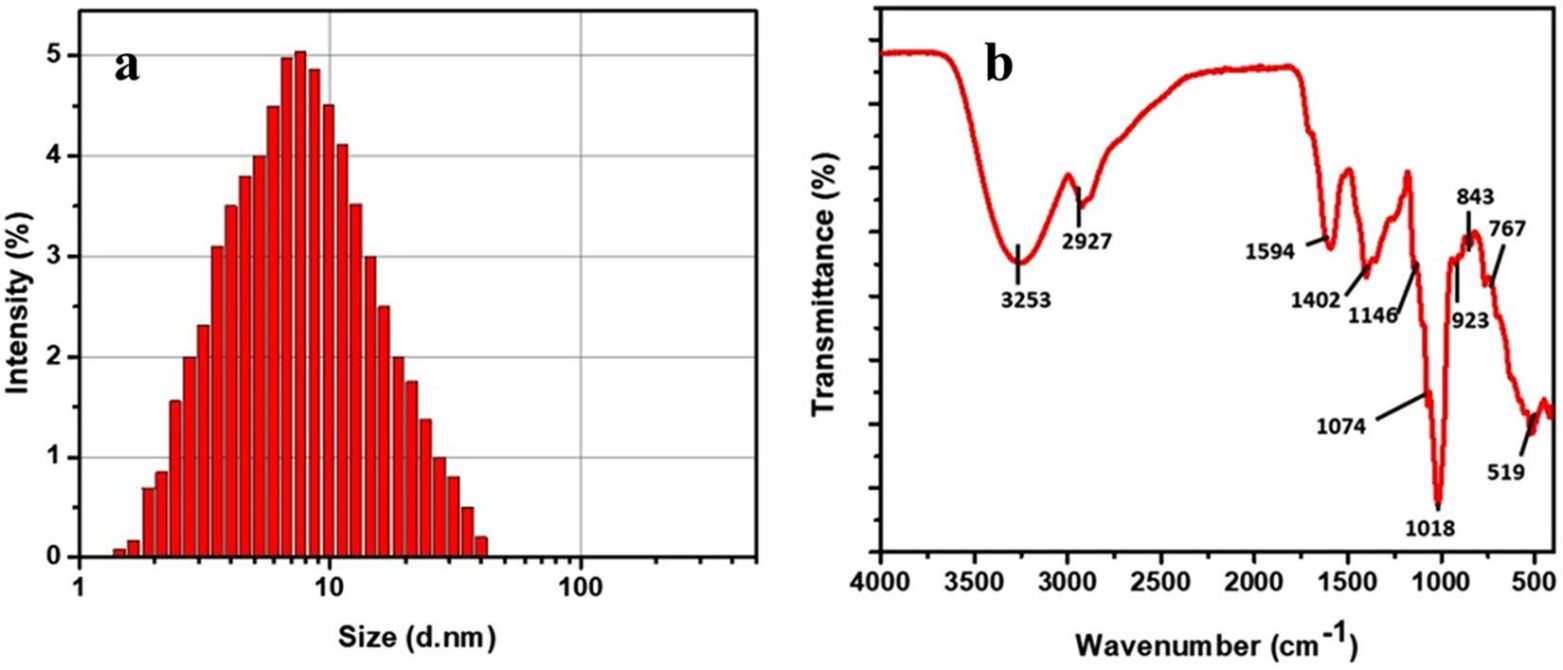
Particle size (**a**) and Fourier-transform infrared spectroscopy (**b**) analysis of the Tv-AgNPs.

The XRD pattern as indicated in Fig. 2e confirmed the natural formation, crystallinity, and purity of Tv-AgNPs in accordance with Bragg reflection of (111), (220), (200), (311). Compared to Joint committee on powder diffraction standards (JCPDS-89–3722), the results are similar to the earlier reports of XRD patterns of silver nanoparticles^42,43^. The EDS and XRD results confirmed the successful synthesis of the silver nanoparticles using the bark extract of *T. vernicifluum*. Furthermore, the PSA analysis revealed the size range of Tv-AgNPs size from 2–40 nm with an average of 12.01 nm (Fig. 3a), which is in agreement with the FETEM results. As indicated by FETEM and PSA, different morphological structures of Tv-AgNPs generated were due to excessive capping or binding of the bark extract of *T. vernicifluum*. Similarly, several earlier works have reported the significant involvement of phytochemicals in the generation and properties of the AgNPs^39,40,44,45^.

The FTIR analysis was performed to determine the capping of functional biomolecules in Tv-AgNPs and the results are depicted in Fig. 3b. There are several stretching vibrations as 3253 (weak O-H stretching, alcohol), 2927 (strong N-H stretching, amine), 1594 (strong C=O stretching, amide), 1402 (week C=C stretching, aromatic), 1146 (medium C-N stretching, amine), 1074 (strong C-O stretching, aromatic ester), 1018 (Strong C-F, fluoro compound), 923 (strong C=C, alkaline), 843 (C-Cl, halo compounds), and 767 (Strong C-H), 519 (C-l). This indicated that the presence of amine, amide, phenolic, alcoholic aromatics from the bark extract of *T. vernicifluum* involved as reducing or capping agent in the synthesis of the Tv-AgNPs and this finds support of earlier reports^32,38,46–48^.

### Cytotoxicity and anti-proliferation assay

**Figure 4:**
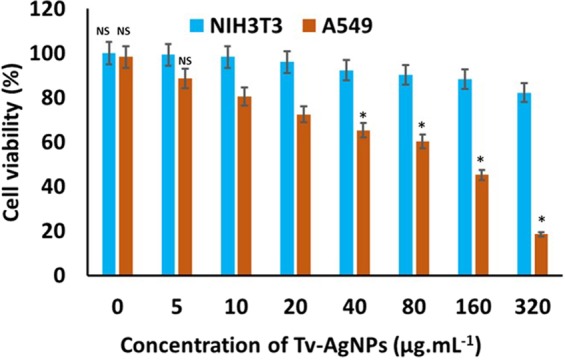
Cytotoxicity and antiproliferative effect of Tv-AgNPs on mouse embryo fibroblast cell line NIH3T3 and human lung carcinoma A549. NS- Not significant with NIH3T3 and A549. *p significantly differs.

Analysis of cytotoxicity of biological materials is essential for the pharmacological trails. Hence, the present study determined the cytotoxicity of Tv-AgNPs by CAM and WST assays. CAM assay revealed that that exposure of negative control with 0.1 M NaOH induced the blood hemorrhage and coagulation, while the distilled water did not cause any irritant reaction. The Tv-AgNPs exposure did not cause any irritant reaction at 50 µg.mL^−1^, but slightly irritant only at 100 µg.mL^−1^ (S.Fig. 1). Further, *in vitro* cytotoxicity assay was performed on mouse embryo NIH3T3 cells and the results indicated that the NIH3T3 cells growth was not significantly reduced with the treatment of Tv-AgNPs at different concentrations (Fig. 4), In addition, AO/EB and DCFH-DA staining showed no cell death and ROS generation respectively with the treatment of Tv-AgNPs (S.Fig. 2). Further, the apoptosis analysis by flow cytometer assay indicated only negligible apoptosis (0.50%) with the treatment of Tv-AgNPs (S.Fig. 3a,b). These results of CAM assay, cell toxicity (WST assay), AO/EB, DCFH-DA staining and flow cytometric (Annexin V FITC/PI) analyses confirmed the non-toxicity of Tv-AgNPs. Similarly, the non-toxicity of phytogenic AgNPs is reported on normal cell lines of renal (MDCK)^49^, epithelial HBL-100^43^, and Human embryonic kidney HEK 293^50^.

**Figure 5:**
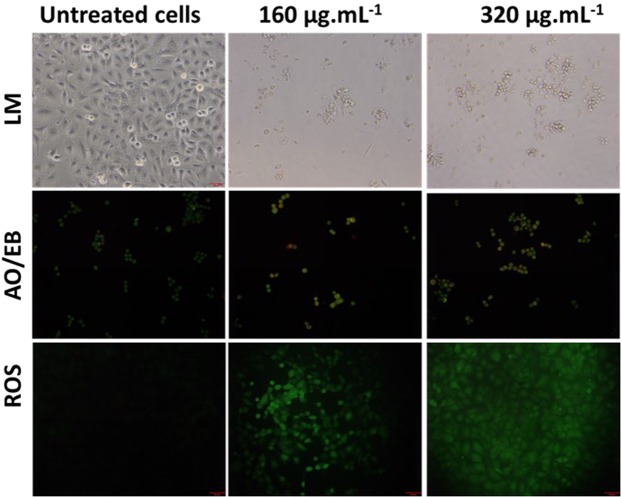
Effect of Tv AgNPs treatments and untreated on cellular morphology changes and reactive oxygen species generation in human lung carcinoma A549.

On the other hand, the treatment of Tv-AgNPs induced the death of human lung cancer cells in A549 cell line at concentration-dependent manner (Fig. 4). About 82.5% of cells were dead in the treatment of Tv-AgNPs. Similarly, Annexin V FITC/PI based apoptosis assay also showed about 95% cell death with treatment of Tv-AgNPs at 320 µg.mL^−1^ (S.Fig. 3c,d). Further, AO/EB, DCFH-DA staining results showed the cell damage and ROS generation at the exposure to 320 µg.mL^−1^ of Tv-AgNPs (Fig. 5). This indicated the smart cancer cells sensing efficiency of Tv-AgNPs in causing cancer cell death through ROS mediated apoptosis in human lung cancer cells, induction of the oxidative stress and reduction of ATP generation required for the cellular energy^51^. Moreover, it is reported that AgNPs trigger the cell apoptosis in human breast cancer cell MCF-7, human lung carcinoma A549, HCT116, HepG2^52^, colon cancer cell line HT-29, SW620 through the interactions with cell organelles including mitochondria, nucleus, proteins, and DNA^53^.

### Antibacterial activity

**Figure 6:**
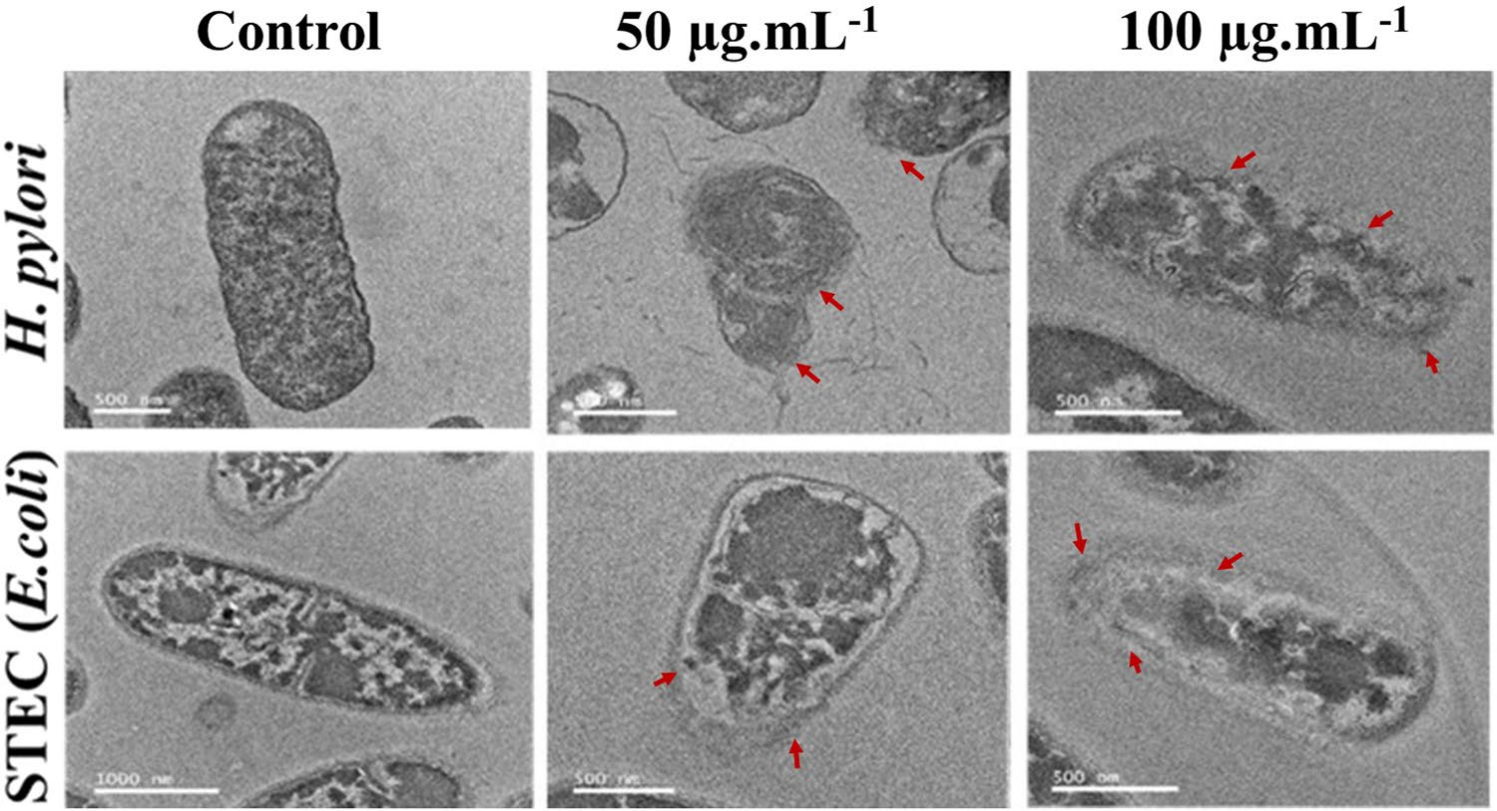
Antibacterial activity; transmission electron microscopic observation of cellular morphology changes in bacterial cells treated or untreated with Tv AgNPs, the red arrow indicates the cell damage.

Silver nanoparticles inhibit the bacterial pathogens by penetrating through the bacterial cell wall and binding with peptidoglycan or lipopolysaccharide, subsequently damaging the bacterial membrane, forming the membrane pits, and inducing the leakage of cellular materials^54–57^. Similarly, the silver nanoparticles synthesized in the present study displayed potent antibacterial activity at the minimal inhibitory concentration of 8.12 µg.mL^−1^ for STEC and 18.14 µg.mL^−1^ for *H. pylori*. Further, the disc diffusion assay revealed that Tv-AgNPs at 100 µg.mL^−1^ displayed the higher zone of inhibition against *H. pylori* (17 mm) and STEC (22 mm) than the standard kanamycin (S.Fig. 4). The bacterial cellular damage and cell disruption due to the treatment of the Tv-AgNPs were observed under TEM, and the images clearly indicated the cell wall damage with elution of cellular inclusions by the treatment of Tv-AgNPs in comparison to untreated cells of *H. pylori* and STEC (Fig. 6). This potential activity is likely due to unique shape and size of Tv-AgNPs for easy penetration into the bacterial cells followed by ROS formation, DNA damage, and cellular membrane damage, growth signalling pathway and tyrosine phosphorylation^42,58–60^. Similarly, silver nanoparticles, synthesised from the *Solanum xanthocarpum* L. and *Peganum harmala* L are reported to significantly inhibit the growth of the *H. pylori*^61,62^.

## Conclusion

The prevention of the microbial infections and biofilm formation by dangerous microbes such as *H. pylori* and STEC is essential for human health. Eco-friendly, cost-effective and green method was attempted to synthesise the Tv-AgNPs using aqueous bark extract of *T. vernicifluum* as reducing or capping agent. The size of Tv-AgNPs ranged from 2–40 nm with anisotropic structure, spherical and oval shape, as revealed by TEM and PSA. The Tv-AgNPs were nontoxicity as confirmed through CAM assay on Egg and cytotoxicity assay in NIH3T3. The small-sized (<40 nm) Tv-AgNPs displayed potential antibacterial, and anti-proliferative activities by inducing the ROS, oxidative stress, DNA division, nucleus damage, and apoptosis in both cancer and bacterial cells. Hence, the Tv-AgNPs deserve for the preparation of biomedical products such as would dressing cloth, and surgical devices.

## Materials and Methods

### Chemicals, bacterial strains, and cell culture

Gram-negative bacterial human pathogens such as *Helicobacter pylori* (MH179988) and *Shigella* toxin producing *Escherichia coli* (MH180008) were received from the laboratory of Professor Deog-Hwan Oh, College of Biotechnology and Bioscience, Kangwon National University, Chuncheon, Republic of Korea. The bacterial strains were preserved in 20% glycerol at −80 °C. The chemicals such as dichlorofluorescein diacetate (DCFH-DA), trypsin, acridine orange Hemi salt (AO), ethidium bromide (EB) were obtained from Sigma Aldrich, Republic of Korea. The cell viability proliferation and cytotoxicity assay kit (EZ-CYTOX water-soluble tetrazolium (WST) (EZ-CyTox)) was purchased from Daeil Lab Service, Republic of Korea. Dulbecco’s modified eagle medium (DMEM), penicillin, streptomycin, and fetal bovine serum (FBS) were procured from Thermo Fishers Scientific Seoul, the Republic of Korea. Dead Cell Apoptosis Kit with Annexin V FITC/PI - for Flow Cytometry was purchased from Invitrogen, Thermo fishers scientific, Republic of Korea. Mueller Hinton Broth was obtained from MB cell, Seoul, Republic of Korea. The cell lines such as mouse embryo fibroblast cell line NIH3T3 and human lung carcinoma A549 were received from the Korean cell line bank (Seoul, Republic of Korea). The bark sample of *Toxicodendron vernicifluum* was collected from Wonju-malgeun-chamott, Wonju city, Republic of Korea.

### Synthesis and characterization of Tv-AgNPs

The bark samples were subjected to the water extraction according to the methods reported earlier with minor modifications^63,64^. The bark extract was prepared by boiling the 5 g of bark samples in 100 ml of distilled H_2_O at 90 °C for 10 min, followed by that the extracts were cooled in room temperature then collected by the centrifugation at 10000 rpm for 20 min. Finally, the extracts were filtered through the Whatman No.1 filter paper and stored in room temperature for further use. For the synthesis of the Tv-AgNPs, 3 mM of AgNO_3_ was dissolved in 10 ml of bark extract at room temperature. The synthesis of Tv-AgNPs was observed by scanning the reaction mixture in a range from 200 to 700 nm using the UV spectrophotometer (Optizen 2120UV, Korea). To analysis the morphology, shape and dispersion characteristics, the Tv-AgNPs were carbon coated in copper grid and then observed under Transmission electron microscopic (TEM, JEOL-JSM 1200EX, Japan) with Energy dispersive X-ray spectroscopy (EDS), X-ray diffractometer (X’pert-pro MPD- PANalytical, Netherland) operated at 40 keV, 40 mA with Cu κα radiation in θ−2θ. The size of Tv-AgNPs was measured using particle size analyzer (PSA, Malvern Mastersizer 2000, Britain). The chemical nature and functional groups present in Tv-AgNPs were analysed by using Fourier-transform infrared spectroscopy (FTIR PerkinElmer Paragon 500, USA).

### Cytotoxicity

Allergic and toxic effect of Tv-AgNPs was tested by using chick embryo chorioallantoic membrane (CAM) assay^65,66^. Cytotoxicity and anti-proliferation effects of Tv-AgNPs were investigated on NIH3T3 and A549 cells respectively using WST assay^67^. Briefly, NIH3T3 or A549 (1 × 10^4^) cells were seeded in 96-well plates containing DMEM or RPMI 1640 medium and allowed in 5% CO_2_ incubator at the humidified environment for overnight to get 80–90% confluence. Then 10 µl of Tv- AgNPs at the different concentrations (0–320 µg.ml^−1^) were added to Tv-AgNPs; after 12 h of exposure the WST1 reagent was added and incubated for 30 min to 4 hours; and then measured the absorbance at 450 nm as per manufacturer’s instructions of WST-1 method. The experiments were conducted in three independent trials with three replicates for each trial and the cell viability was determined. Further, the effect of Tv-AgNPs treatment on NIH3T3 and A549 cells was analysed for morphological changes^1,68^. The DCFH-DA stain assay was used to measure the ROS generation at an excitation of 495 nm and emission of 529 nm. Apoptosis was observed by AO/EB staining assay^69^ and images were taken using the fluorescence microscope (Olympus, CKX53 culture microscope, Japan).

### *In vitro* antibacterial assay

Effect of Tv-AgNPs on the eradication of *H. pylori* and Shigella toxin producing *Escherichia coli* (STEC) was analyzed using the microdilution method (Clinical and laboratory standard institute, CLSI). For the elucidation of minimal inhibitory concentration (MIC), the STEC was grown in Mueller Hinton Broth (MHB) and *H. pylori* in brain heart infusion (BHI) broth in a rotary shaker at 180 rpm at 37 °C for 24 h. The bacterial suspension (10^9^ CFU.ml^−1^) was dispensed in 96-well (Costar) plates containing different concentrations of Tv-AgNPs (0.1–12.5 µg.ml^−1^). The un-inoculated MHB and untreated bacterial cells were used as negative and positive controls respectively and optical density was measured at 600 nm^70^. Tv-AgNPs induced bacterial cell disruption was observed using high-resolution transmission electron microscopy (HRTEM)^71^. For HRTEM analysis, the MIC of PDK-CE was treated to *H. pylori* for 24 h at 37 °C. After the treatment period, the cells were collected by centrifugation, then fixed them with 4% glutaraldehyde (v/v) for 2 h and the cells were dehydrated by acetone (70%). Finally, the cellular changes were observed using the HRTEM (JEOL-2010, Japan).

## Supplementary information

Unveiling the potentials of biocompatible silver nanoparticles on human lung carcinoma A549 cells and Helicobacter pylori
