# Supplementary material for: Unveiling the potentials of biocompatible silver nanoparticles on human lung carcinoma A549 cells and Helicobacter pylori
Source: Sci Rep. 2019 Apr 8;9:5787. doi: 10.1038/s41598-019-42112-1 (PMC6453883; doi:10.1038/s41598-019-42112-1)
Supplement: Supplementary file 1 — Unveiling the potentials of biocompatible silver nanoparticles on human lung carcinoma A549 cells and Helicobacter pylori [file 41598_2019_42112_MOESM1_ESM.doc]

**Unveiling the potentials of biocompatible silver nanoparticles on human lung carcinoma A549 cells and *Helicobacter pylori***

**SUPPLEMENTARY INFORMATION**


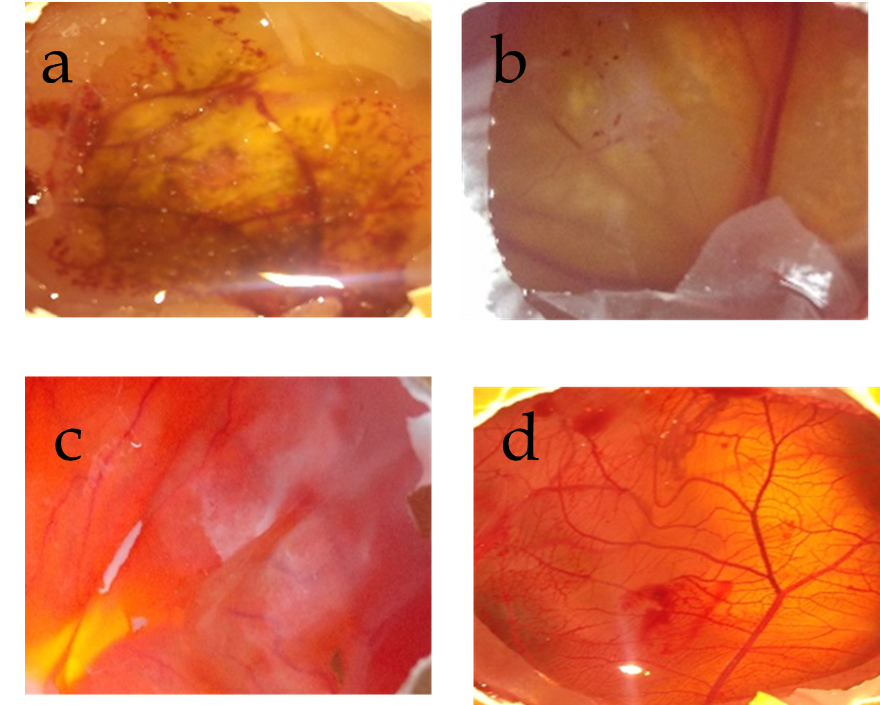


S.Fig.1. Toxicological evaluation of Tv-AgNPs by chick chorioallantoic membrane (CAM) assay. 0.1 M NaOH (a), distilled water (b), 50 µg^.^mL^-1^ of Tv- AgNPs (c), 100 µg^.^mL^-1^ of Tv- AgNPs


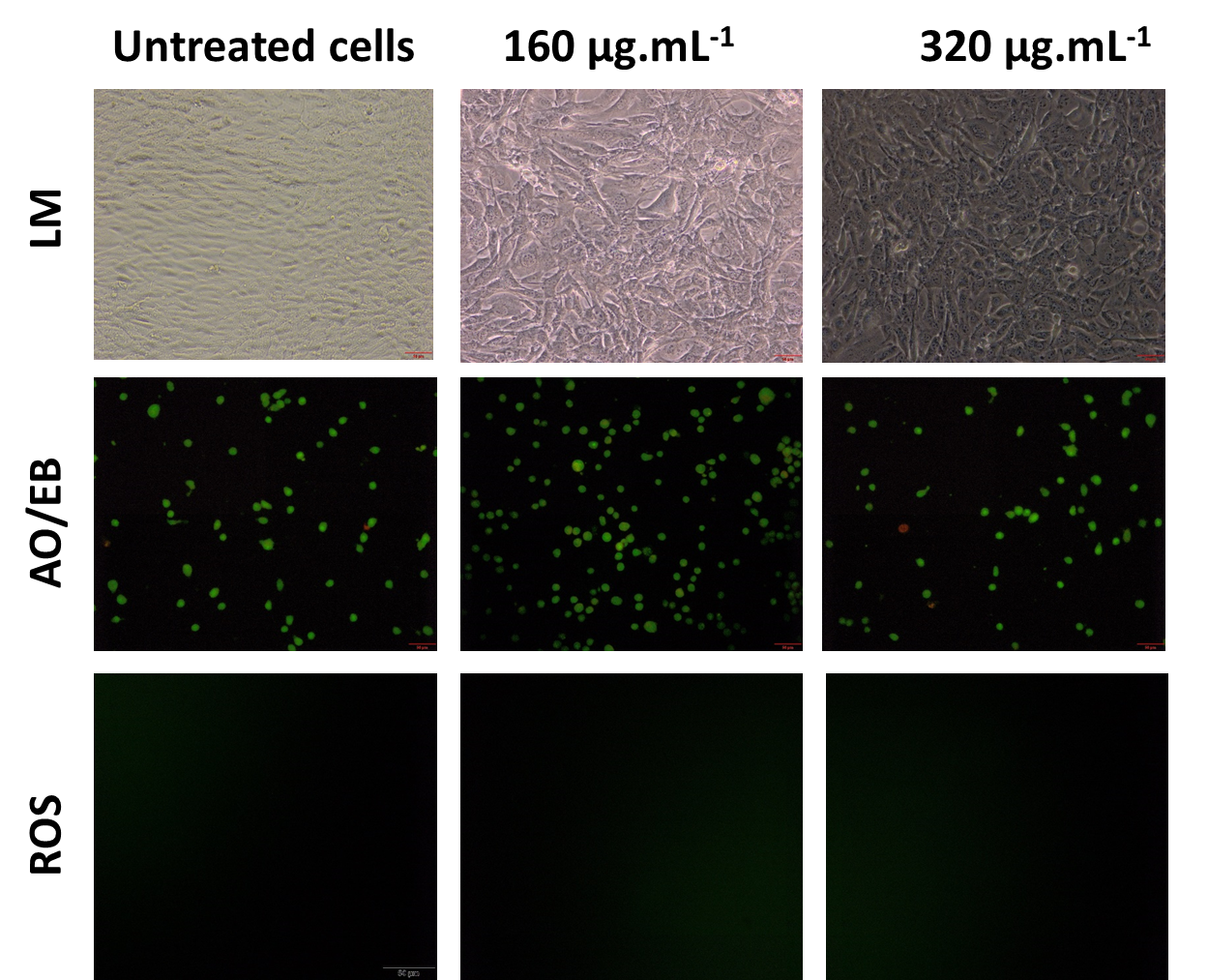


S.Fig.2. Effect of Tv AgNPs treatments and untreated control on cellular morphology changes and reactive oxygen species generation in mouse embryo fibroblast cell line NIH3T3. LM light microscopy, ROS- reactive oxygen species, AO/EB-Acridine orange/ Ethidium bromide.


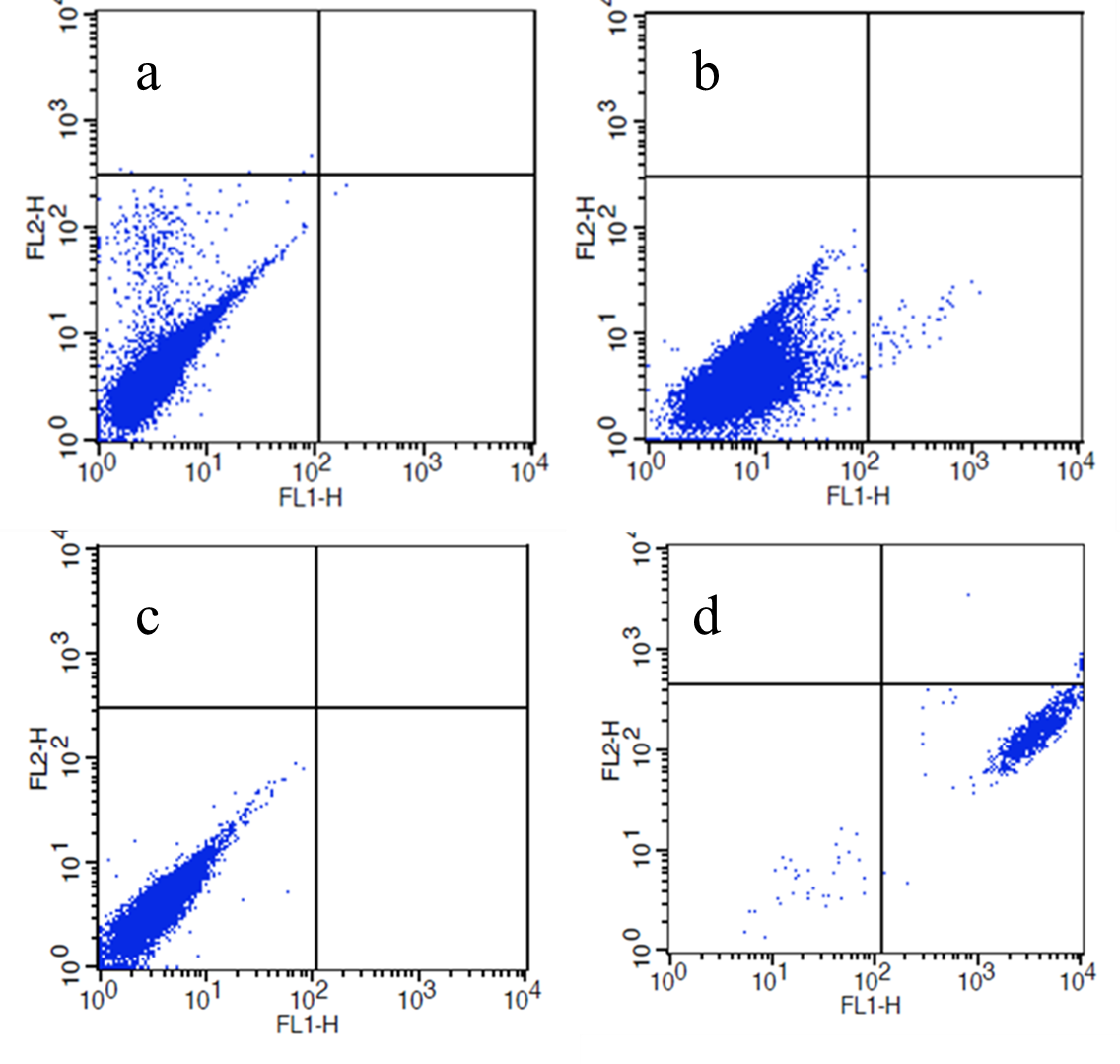


S.Fig.3. Flow cytometer-based analysis of the cell viability. Untreated mouse embryo fibroblast cell line NIH3T3 (a), mouse embryo fibroblast cell line NIH3T3 cells treated with Tv-AgNPs (b), Untreated A549 cells (c), A549 cells treated with Tv-AgNPs (d)


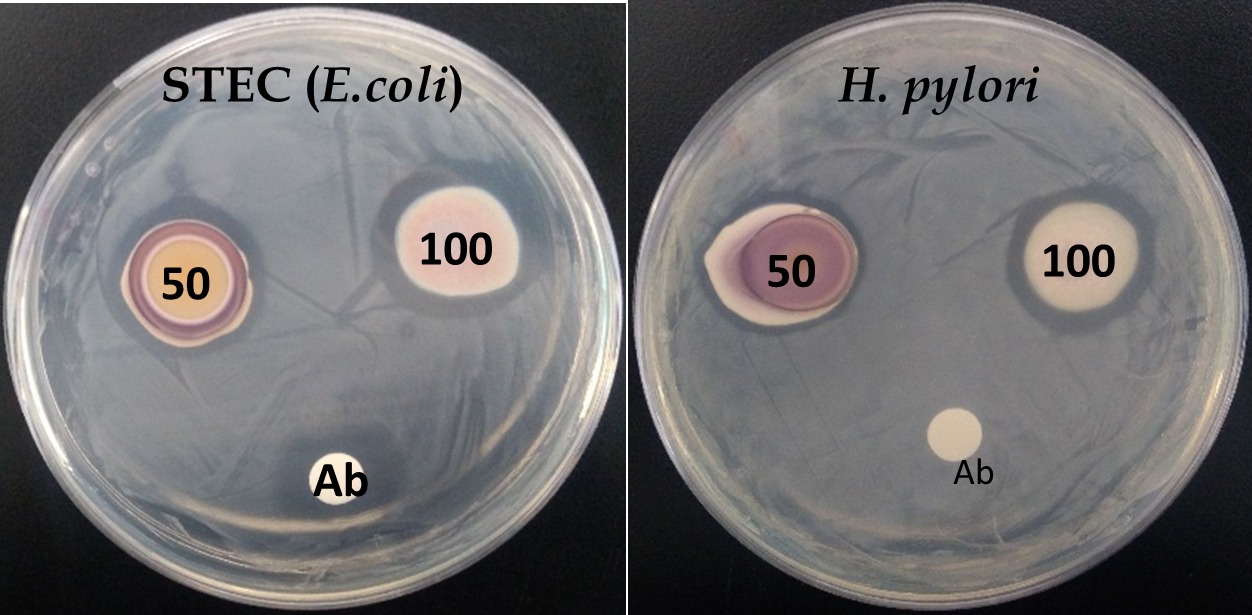


S.Fig.4. Antibacterial activity Tv-AgNPs (50, 100µg.mL^-1^) synthesized from bark extract of *Toxicodendron vernicifluum* (Ab- Kanamycin (10 µg)
